# 分子印迹聚合物的设计合成及其在医学诊疗与生物催化中的应用

**DOI:** 10.3724/SP.J.1123.2025.06013

**Published:** 2026-02-08

**Authors:** Qingmei SONG, Xinhao LI, Yongqin LYU

**Affiliations:** 北京化工大学生命科学与技术学院，有机无机复合材料全国重点实验室，国家能源生物炼制研发中心，绿色 化学品生物制造北京市重点实验室，教育部生物能源国际合作联合实验室，北京 100029; College of Life Science and Technology，Beijing University of Chemical Technology，State Key Laboratory of Organic-Inorganic Composites，National Energy R&D Center for Biorefinery，Beijing Key Laboratory of Green Chemicals Biomanufacturing，International Joint Bioenergy Laboratory of Ministry of Education，Beijing 100029，China

**Keywords:** 分子印迹聚合物, 智能响应材料, 生物催化, 医学诊疗, 高通量筛选与精准识别, molecular imprinting polymers（MIP）, stimuli-responsive materials, biocatalysis, medical diagnosis and therapy, high-throughput screening and specific molecular recognition

## Abstract

分子印迹技术（MIT）因设计灵活、适用广泛，已在疾病诊断、环境监测与食品安全等领域展现出重要应用潜力。然而，传统本体聚合法仍存在传质效率低、模板残留严重及大分子印迹效率不足等问题，同时受制于成本、标准化及跨学科融合等因素，限制了MIT的规模化应用。针对上述问题，本团队围绕功能单体的高通量筛选与识别机制解析，建立了标准化功能单体库，并结合粗粒化模拟，实现了复杂体系中印迹聚合物（MIPs）的精准设计与可控合成。通过技术路径优化与材料体系升级，开发出高效固相模板表面印迹及具温度/pH响应特性的智能印迹材料，其结合亲和力显著提升，平衡解离常数（*K*_D_）可达10^-12^ mol/L，较非印迹聚合物提升逾万倍。在应用层面，团队构建了MIPs的多维交叉体系：在生物分离纯化中实现复杂体系中蛋白质的高效富集；在生物催化中搭建双酶级联系统以提升催化效率与酶活复性；在生物医学方向开发出皮克（pg）级灵敏度的生物标志物检测与超低浓度肿瘤细胞（5个细胞/mL全血）识别平台，拓展至肿瘤诊疗一体化与微生物群落干预等前沿领域。本文综述了近5年MIT在生物医学与生物催化领域的研究进展，系统总结了本团队在MIPs理性设计、制备方法及交叉应用方面的成果，并展望其在智能响应材料与集成化诊疗系统中的发展方向。

## Abstract (en)

Molecularly imprinted technology （MIT） represents an advanced synthetic strategy that emulates biological recognition mechanisms， such as antigen-antibody or enzyme-substrate interactions， by creating three-dimensional cavity-like structures through the directional assembly of functional monomers around a template molecule. This process generates spatial and functional complementarity， enabling highly selective recognition of target species. Molecularly imprinted polymers （MIPs）， often described as “synthetic antibodies”， overcome the intrinsic limitations of natural biomolecules by offering superior selectivity， robustness， cost-effectiveness， and structural tunability. These features position MIPs as promising alternatives to natural antibodies in targeted sensing and drug delivery， with broad applications across biomedical， environmental， and pharmaceutical domains， including pollutant detection， and food safety monitoring. Despite substantial progress， key challenges remain， such as uneven imprinting layers， template residue， and limited aqueous compatibility in macromolecular imprinting. Furthermore， issues of industrial scalability， unclear recognition mechanisms， and insufficient integration with emerging fields such as microfluidics and artificial intelligence have hindered large-scale translation. In recent years， our research team has systematically advanced MIT through a tri-dimensional strategy encompassing high-throughput monomer screening， mechanistic elucidation of molecular recognition， and directional assembly of functional units. By establishing a standardized monomer library and integrating molecular dynamics simulations， we achieved precise material design under complex conditions. Through process optimization and material innovation， we developed a highly efficient solid-phase surface imprinting method that enables the fabrication of smart MIPs with stimuli-responsive properties （e.g.， temperature and pH）. These MIPs exhibit markedly enhanced binding affinity， with equilibrium dissociation constant （*K*_D_） reaching 10^-12^ mol/L， over four orders of magnitude higher than those of non-imprinted polymers （NIPs）. Building on these advances， we established cross-disciplinary application platforms， including affinity-based protein separation and purification systems capable of efficient dual-enzyme cascade immobilization and inactivated enzyme renaturation. In the biomedical domain， we developed ultrasensitive biosensing methods achieving picogram-level detection of heart failure biomarkers and single-digit （≈5 cells/mL） detection of cancer cells in whole blood， extending these methods toward integrated tumor theranostics and microbial community regulation. This paper comprehensively summarizes our team’s recent innovations in the rational design， functionalized fabrication， and cross-disciplinary applications of MIPs， spanning biosensing， biocatalysis， and biomedical diagnostics/therapeutics， while contextualizing these within the latest global advances in biomedicine and catalysis. Looking forward， we identify three strategic research frontiers for next-generation MIT. （i） Smart responsive material systems： design MIPs capable of multi-stimuli responsiveness （e.g.， magnetic， photothermal， and pH cues） to enable programmable drug release， real-time signal monitoring， and dynamic feedback regulation. （ii） Quantitative modeling of dynamic recognition： establish multi-scale theoretical frameworks to elucidate coupling between cavity flexibility and target conformational dynamics， guiding structure optimization and function-oriented design of adaptive MIPs. （iii） Integrated intelligent theranostic platforms： integrate microfluidics and biomimetic recognition modules into closed-loop systems capable of biomarker detection， targeted delivery， and real-time therapeutic feedback， bridging the gap between in vitro sensing and in vivo precision intervention. Synergistic advancement along these trajectories will empower MIT to transcend its role as a “static recognition material” and evolve into an intelligent， adaptive， and systematic biomedical platform. Such evolution will accelerate the translation of MIT innovations from laboratory to clinic and industry， propelling progress in personalized medicine， point-of-care diagnostics， and synthetic biology， and yielding profound scientific and societal impact.

分子印迹技术（MIT）是一种模拟天然分子识别机制（如抗原-抗体特异性结合、酶-底物识别）的人工智能识别方法。其核心原理在于以目标分子为模板，通过功能单体的定向组装与交联聚合，构建出具备空间互补性与功能基团匹配性的三维识别空腔，从而赋予材料类生物系统的高选择性识别能力。这种以“仿生蓝图”为基础的分子设计，使MIT被誉为“合成抗体”或“分子锁钥识别系统”。

MIT的理论基础可追溯至1940年Pauling提出的抗原-抗体专一识别模型^［1］^。1972年，率先采用共价印迹法在有机聚合物中成功构建“记忆空腔”，开启了MIT的实验应用^［2］^。随后，Mosbach团队于1993年提出非共价印迹策略，推动了该领域技术体系的丰富化，包括共价/非共价协同印迹、金属螯合印迹等方法相继出现^［3］^。经过数十年的发展，MIT在多个领域实现了从理论向应用的跃迁：生物医药领域可用于固相萃取、化学传感与手性药物分离^［4］^；环境监测中实现了痕量污染物的高灵敏检测；食品安全方面可用于残留筛查；药物递送中则用于构建可控释放系统^［5］^。近年来，随着纳米技术与计算化学的融合，MIT在智能响应材料、高精度识别等方向取得突破，推动其向智能化与精准化方向演进。

分子印迹聚合物（MIPs）作为MIT的典型产物，凭借其设计灵活性、高特异性识别能力以及对多类目标物的广泛适应性，正成为生物识别领域的核心材料^［6］^。相较于天然抗体和酶类，MIPs具备更优的稳定性和成本优势。通过对模板分子、功能单体及交联剂的合理搭配，可精准调控其物理化学性能，实现对小分子、金属离子甚至蛋白质等多尺度目标物的识别^［7，8］^。当前，MIPs作为“合成抗体”在生物医学应用领域展现出巨大的应用潜力，已成为国际研究的前沿热点。其核心优势在于可突破天然抗体稳定性差、成本高、制备难度大的限制，实现特定靶标的理性设计与大规模、低成本制备。近年来，国际前沿研究广泛聚焦于表位印迹策略，通过选择靶蛋白的关键结构片段（如疾病标志物片段、病毒刺突蛋白表位）作为模板，提高MIPs对复杂蛋白质的亲和力与识别选择性。该策略已在多项关键应用中取得突破性进展，如构建具备皮克（pg）级超高灵敏度的生物传感器，以及开发用于靶向干预的印迹材料（如阻断病毒入侵、抑制肿瘤转移等）^［9］^。尽管取得重要进展，MIPs在生物医学应用中仍面临多项关键瓶颈：其一，生物大分子天然具有构象柔性和动态变化，导致在印迹过程中难以形成高度保真、稳定的识别位点，从而限制了识别精度的进一步提升；其二，水相环境中普遍存在的非特异性吸附效应，严重影响了MIPs在复杂生理基质（如全血、血清）中的识别准确性和应用稳定性；其三，高亲和力MIPs的规模化、标准化和可重复制备工艺尚不成熟，成为制约其产业化和临床转化的核心障碍。上述挑战凸显出当前研究亟需在两个维度取得突破：一方面，深化对MIPs分子识别机制的基础理解，特别是在动力学行为、限域识别和多点相互作用等层面的精准表征；另一方面，加强面向实际应用场景的材料工程创新，推动印迹材料在复杂生物环境中的性能优化与功能集成化发展^［10-12］^。

针对上述关键难点与技术瓶颈，本研究团队围绕MIPs的设计原理与应用转化开展了系统性的研究工作。通过构建高通量功能单体筛选平台与标准化功能单体数据库，并结合粗粒化模拟策略，建立了模板分子-功能单体间的作用模型，从而实现了在复杂环境中对目标生物大分子的高特异性识别材料的精准构建与可控合成。在技术路径上，我们突破传统本体聚合法的局限，开发出高效的固相模板表面印迹技术，以及具备温度/pH等多种刺激响应特性的智能印迹材料，显著提高了MIPs对靶标分子的结合亲和力，其平衡解离常数*K*_D_值达10^-12^ mol/L，较非印迹聚合物（NIPs）提升逾万倍。在跨领域应用拓展方面，团队构建了多元化的功能平台体系：在生物分离纯化领域，MIPs被用作高选择性的亲和介质，实现了复杂样本中目标蛋白的高效分离与富集；在微纳尺度平台中，基于MIPs构建的双酶级联催化系统有效提升了酶的稳定性与催化效率，成功实现对失活酶的功能复性与再利用。在生物医学方向，团队开发出一系列MIPs基础上的高灵敏检测平台，已实现对肿瘤标志物、肿瘤细胞及病原微生物的精准识别，检测灵敏度达到pg级别；此外，相关平台亦在肿瘤诊疗一体化、微生物群落调控等新兴领域展现出良好的拓展潜力与技术适配性。

综上所述，本文结合近5年MIT在生物医学与生物催化领域的前沿研究进展，系统梳理了本研究团队近年来在分子印迹材料的理性设计、功能化制备以及在生物传感、生物催化和医学诊疗等交叉应用方面取得的创新成果。结合产业发展趋势与实际应用需求，通过多维技术路径的协同推进，MIT有望突破“静态识别材料”的应用边界，向智能化、动态化、系统化的下一代生物医学平台转型。这一演进不仅将加速MIT从实验室创新向临床与产业应用的转化进程，也将为个性化医疗、即时诊断及合成生物学等前沿领域注入新的技术动能，产生深远的科学与社会影响。

## 1 分子印迹聚合物的理性设计

MIPs作为人工合成的“抗体替代品”，其设计理念与天然抗体的分子识别机制在本质上高度相似。天然抗体由多种氨基酸单体以特定序列聚合而成，形成具有三维空间构象的聚酰胺结构（即蛋白质），该构象依赖氢键、离子键、疏水效应以及二硫键等弱相互作用力共同维持。这一结构特征使抗体能够高选择性地识别并结合其特定抗原^［5］^。这种“结构-功能”关系也为MIPs的理性设计提供了启示。在MIPs合成中，通过功能单体（如乙烯基类氨基酸化合物）与模板分子间的协同作用，可构建出在空间构型与化学环境上均与目标分子互补的结合位点。

MIPs的合成过程通常包括模板分子、功能单体、交联剂、引发剂及溶剂等多个关键组分。在预聚合阶段，模板分子与功能单体间形成可逆的相互作用复合物。随后，通过交联剂引发聚合反应，将复合物结构固定于聚合物基体中。模板去除后，即形成具有分子识别能力的印迹空腔。已有研究表明，功能单体的种类、比例及其与模板的相互作用强度，是决定印迹效果的核心因素。然而，传统的单体组合筛选依赖实验迭代，费时且效率低。

为加快MIPs配方优化过程，多种高通量筛选方法被提出，包括高效液相色谱（HPLC）、流动注射分析、计算机模拟及酶联免疫吸附法（ELISA）等^［13-17］^。尽管这些策略在提高筛选效率方面取得了一定进展，但如何建立更加系统且普适的高通量筛选体系，依然是该领域的重要挑战。基于此，本课题组^［18］^提出了一种新型的高通量筛选策略，用于从配方库中快速筛选出具有高亲和力和高选择性的最佳MIPs（图1）。具体而言，我们选取了带有不同电荷性质、中性基团以及疏水性的功能单体，采用沉淀聚合法在96孔板中微量并行合成了超过100种非印迹聚合物微阵列，实现快速筛选。以表皮生长因子受体（EGFR）的一个短肽片段（表位氨基酸420~431：NITSLGLRSLKE）作为模板分子，使用荧光素标记的该肽段对聚合物微阵列进行结合能力测试。通过基于荧光强度的评价体系，我们成功筛选出对EGFR表位具有极高亲和力的MIPs配方，获得的MIP对EGFR结合的平衡解离常数*K*_D_值为3.7×10^-12^ mol/L。此外，该MIPs可在低至5个细胞/mL的全血样本中特异性识别肿瘤细胞，并通过体内荧光成像验证了其优异的靶向能力，展示出其在早期诊断和靶向治疗中的应用前景。

**图1:**
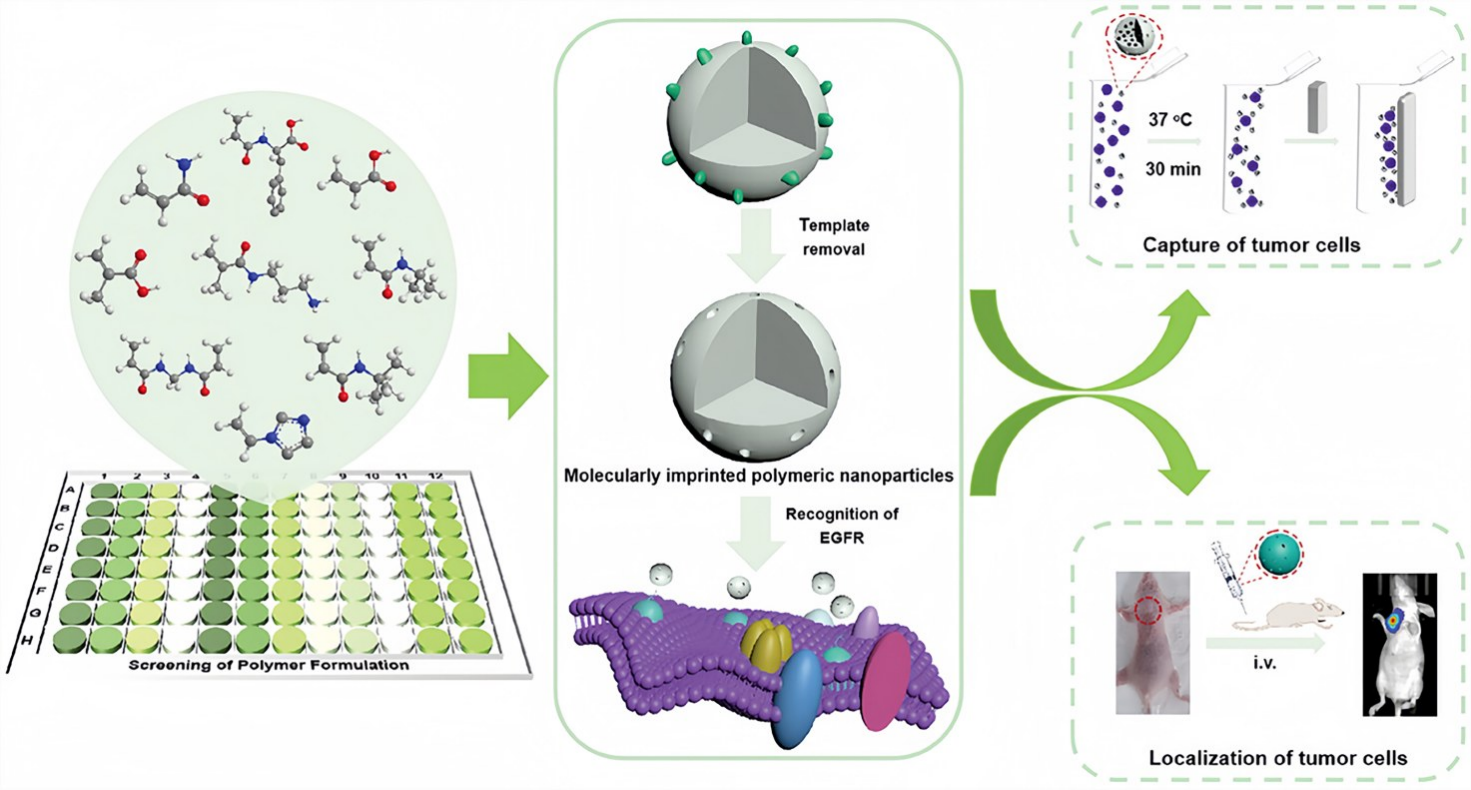
EGFR表位多肽（420~431： NITSLGLRSLKE）作为模板分子，通过高通量筛选策略获得最佳聚合物配方合成的MIPs示意图^［18］^

MIPs识别特异性来源于其“锁-钥匙”式的印迹空腔结构。不同的作用力机制对识别性能影响各异：氢键可增强极性分子的选择性，*π-π*堆积作用有助于识别芳香结构，位阻效应则高度依赖于聚合物交联度和空腔结构的精细调控^［19］^。然而，尽管已有大量实验探索不同印迹机制，目前仍缺乏对模板-共聚物纳米体系协同识别机制的系统理解。

为深入揭示MIPs识别机制的微观本质，本课题组^［20］^构建了一个用于模拟蛋白质标志物与共聚物相互作用的粗粒化分子动力学（CG-MD）模型。在该模型中，选择结构已知且在肿瘤细胞中表达升高的波形蛋白二聚体作为代表性生物标志物，系统研究其与一系列随机共聚物链之间的相互作用（图2）。模拟结果表明，波形蛋白二聚体表面在电荷分布与疏水性方面呈现显著的各向异性，导致不同共聚物在其表面的吸附呈现出非均匀性。多个共聚物链在不同区域的结合存在能量异质性，并表现出明显的协同吸附效应。这种吸附行为不仅依赖于共聚物的组成，还受到其电荷密度与疏水性程度的双重调控。具体而言，当共聚物疏水性较低时，适度增加电荷密度可有效提升其在蛋白质表面的覆盖率；而对于疏水性较高的共聚物，电荷增加反而可能干扰其吸附稳定性，导致整体覆盖率下降。上述现象可归因于波形蛋白二聚体表面多样化功能域的空间分布及其尺寸特征对不同共聚物的选择性结合能力。为进一步量化这些吸附行为，我们基于覆盖度依赖的Langmuir模型，建立了一套模拟策略，通过拟合模拟得到的结合等温线，成功描绘出共聚物与蛋白之间的自由能变化全貌。值得强调的是，该模拟结果与生物层干涉技术（BLI）实验数据高度一致，验证了所提出模型的准确性与可靠性。本研究从分子层面揭示了共聚物与蛋白质之间复杂吸附行为的能量驱动机制，为今后针对特定蛋白靶标的MIPs设计提供了可量化的理论依据。

**图2:**
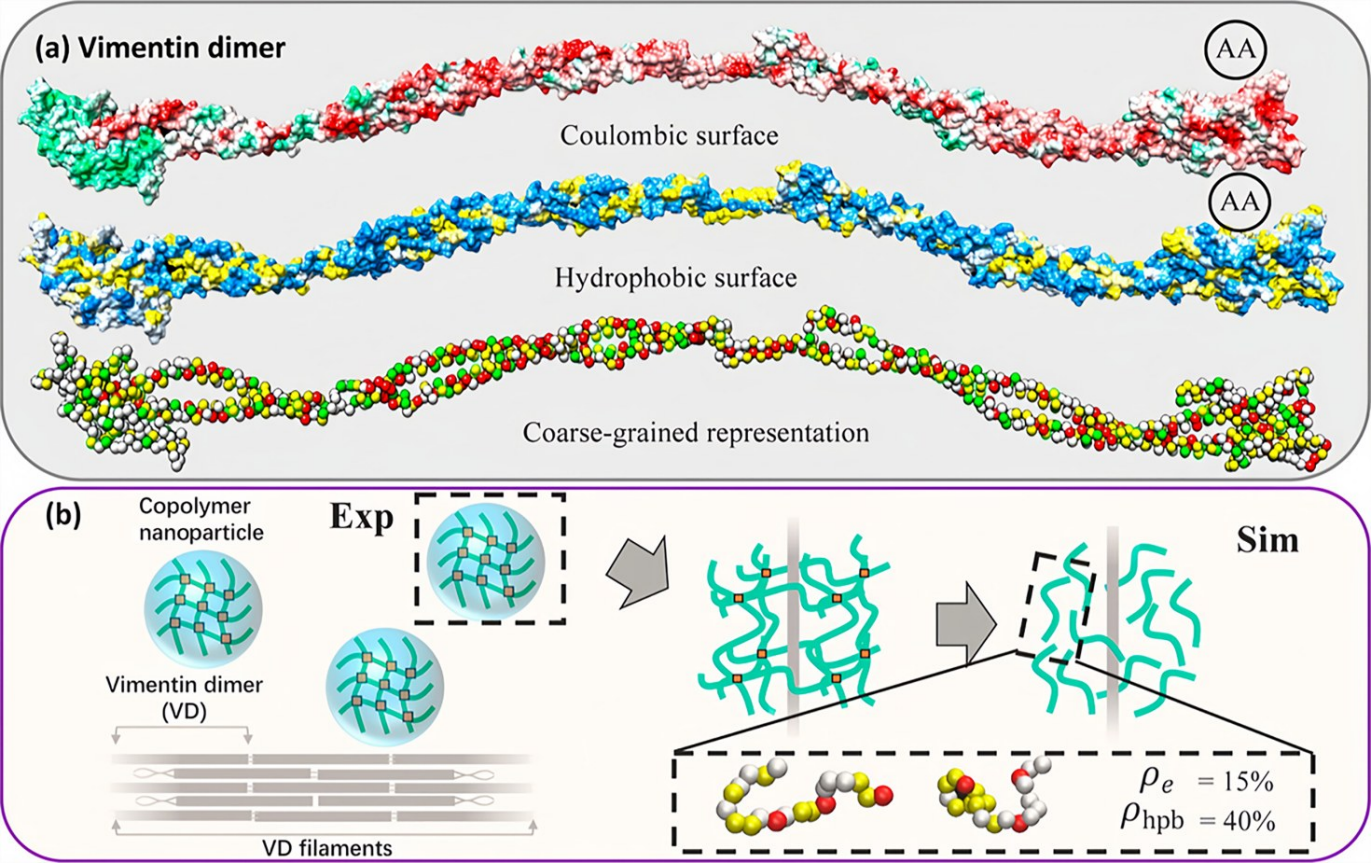
（a）波形蛋白二聚体的全原子结构；（b）波形蛋白二聚体与交联共聚物网络相互作用的示意图^［20］^

在单体设计与筛选方面，国际研究者正致力于开发更智能、通用的策略，以突破MIPs传统依赖经验与试错的局限。值得强调的是，计算模拟技术正由辅助工具迅速演变为MIPs理性设计的核心驱动力，尤其在高性能功能单体的筛选与生物大分子印迹领域中展现出显著优势。通过分子对接（MD）与分子动力学模拟（MDS），研究者可精准预测功能单体与蛋白质等生物大分子模板之间的结合模式、结合能及其在复杂溶剂环境（如水相）中的相互作用稳定性，从而高效指导高亲和力、高选择性的单体虚拟筛选，显著降低传统“试错法”的盲目性^［21］^。针对生物大分子构象柔性高、表面识别位点异质性强的特点，前沿研究引入CG-MD、增强采样（enhanced sampling）等技术，以模拟其动态结构变化与水合效应，辅助构建能特异识别特定构象或表位的印迹空腔^［22］^。此外，机器学习与高通量虚拟筛选平台的深度融合，正在加速建立单体-模板相互作用数据库与预测模型，为实现基于目标蛋白结构特征的“先导单体”设计提供有力支撑^［23］^。上述进展正推动MIPs设计范式由经验驱动的“试错型”向数据驱动的“预测型”根本转变。

## 2 生物大分子印迹策略的演进：从全蛋白印迹、表位印迹到固相模板印迹

### 2.1 全蛋白印迹：从构象匹配到功能重塑

蛋白质在生命活动中承担核心功能，其高度复杂的结构和多样的功能特性使其成为MIPs研究的重要对象。蛋白质印迹材料凭借其特异性识别能力，已广泛应用于蛋白质分离纯化、疾病标志物检测、生物成像及靶向治疗等多个领域，展现出巨大的应用潜力。

作为蛋白质分子印迹的初始策略，全蛋白印迹通过在聚合物基体中构建与目标蛋白空间构象互补的三维空腔，实现了对整蛋白分子的选择性识别。该方法依赖于功能单体与模板蛋白之间的自组装作用，可形成具有空间结构互补和多点相互作用的结合位点，其分子识别特异性可媲美天然抗原-抗体或配体-受体的分子互作^［24］^。

本课题组在该领域开展了系统性研究，尤其在提升酶类蛋白的催化活性和稳定性方面取得了突破性进展。酶作为自然界中效率最高的生物催化剂之一，具备高度区域和化学选择性，可在温和条件下高效催化特定生化反应。然而，一旦脱离细胞内复杂且精细调控的微环境，多数酶的结构稳定性和催化活性会显著下降^［25，26］^。为此，稳定化酶的策略成为研究热点。传统固定化方法虽可提升操作稳定性并实现酶的回收利用，但固定过程常对酶的构象造成干扰，进而影响其催化活性。针对这一瓶颈，本课题组提出了具有原创性的“物理印迹”策略^［27］^，以酶分子为模板，在金属有机框架材料（MOF）内构建出尺寸与形状精准匹配的印迹腔体（图3）。该方法在MOF合成过程中引入目标酶分子，诱导金属节点与有机配体组装形成限域空腔，随后通过高温煅烧去除模板，保留结构精确互补的印迹位点。所构建的印迹腔室能高度选择性地识别其模板酶分子，显著提升固相酶的催化性能与稳定性。实验证明，该策略制备的固相酶在催化活性上可达游离酶的16.7倍，催化效率（*k*_cat_/*K*_m_）提升至14.1倍，均为目前文献报道中的最高水平。更为显著的是，该固相酶在95 ℃高温、pH=2或pH=10极端pH条件下以及甲醇/丙酮等有机溶剂中仍保持65%以上的残余酶活性；连续重复使用18次后，残余酶活性仍高于80%。通过固态紫外-可见吸收光谱（UV-vis）、电子顺磁共振（EPR）、傅里叶变换红外（FTIR）和固态核磁共振（NMR）等多种表征手段，我们揭示了印迹微环境提升酶性能的机制：物理印迹腔室通过限制酶的折叠动力学、增强辅因子配位效率，从而在限域空间中重构活性位点、稳定酶的高活性构象。

**图3:**
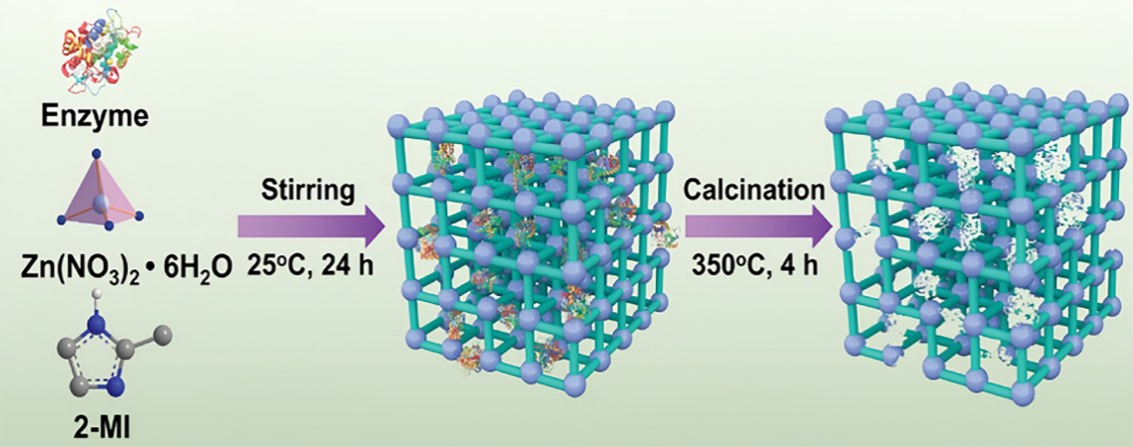
基于“物理印迹”在MOF中构建与酶分子尺寸和形状匹配的“印迹腔室”^［27］^

然而，尽管全蛋白印迹可实现对复杂蛋白质的识别，其仍面临诸多挑战：蛋白质在印迹过程中的构象易发生不可逆变化，导致识别位点结构畸变；同时，大分子模板的使用导致洗脱困难、产率不高，且对模板蛋白的高依赖性使其成本上升、难以实现规模化应用。

因此，尽管全蛋白印迹为分子识别提供了初步解决方案，但其结构可控性与实用性仍有限。针对这些问题，进一步发展更为稳定、灵活、低成本且结构精准的印迹策略，成为推动MIPs从实验室走向实际应用的关键。

### 2.2 表位印迹：突破大分子靶标识别的策略创新

表位印迹（epitope imprinting）技术最初由Rachkov与Minoura提出，旨在弥补稀有蛋白质模板难以获得的难题。该策略通过以特异性短肽片段作为模板，模拟完整抗原的关键识别位点，从而构建具备精准分子识别能力的MIPs材料。这一过程模拟了天然抗体识别抗原表位（无论是构象表位还是线性表位）的机制，其本质依赖于非共价作用力构建的互补三维空间结构^［7］^。近年来，表位筛选技术研究取得显著突破。通过结合计算模拟（如AlphaFold预测蛋白构象）与机器学习辅助设计，研究者可精准定位靶蛋白的高亲和力表位，显著提升印迹效率。例如，Bossi团队^［28］^提出基于表位结构的MDS策略，指导功能单体选择，解决了蛋白构象柔性表位的印迹难题。

尽管表位印迹技术在蛋白质识别方面取得重要进展，但对于更高阶的复杂靶标，如完整细胞，传统的直接印迹方式在实践中面临巨大挑战。细胞本身结构复杂、柔性强、稳定性差，这使得其作为印迹模板时容易发生构象改变甚至变性，从而限制了印迹精度和材料稳定性。为解决上述问题，研究者们逐渐转向间接印迹策略，尤其是针对细胞表面结构中的特异性组分（如膜蛋白、脂多糖（LPS）、糖类、磷脂分子等）进行印迹，以期在保留识别特异性的同时简化操作流程。该方法不仅在分子层面提升了识别的精准性，也显著增强了材料的制备可控性与重现性。例如，微生物（包括细菌、真菌、病毒与微藻）作为一类重要的细胞模型，其识别与检测对于感染性疾病的早期诊断和流行控制具有重要意义^［11］^。革兰阴性菌外膜的主要成分LPS是典型的免疫刺激因子，也是潜在的高选择性识别靶点。本课题组^［29］^以革兰阴性菌的特异性表面标志物LPS为印迹模板，构建了新型人工合成受体（图4）。该受体通过96孔板微阵列筛选获得，并对目标LPS展现出超高亲和力。在PBS缓冲液（10 mmol/L，pH=7.2，25 ℃）中，我们利用BLI检测了MIP/NIP对LPS的结合动力学。以固定生物素化LPS（50 μg/mL）的生物传感器为固相载体，测定不同浓度MIP/NIP的结合响应值。通过1∶1结合模型拟合动力学曲线，获得MIP对LPS的结合平衡解离常数*K*_D_为2.9×10^-12^ mol/L，是NIP的18 897倍，能在全血环境下实现对特定细菌的精准识别，检测灵敏度可达10个细胞/mL。通过在该类人工受体中引入光热转换材料金纳米棒，进一步实现了在近红外照射下的选择性细菌灭活功能，为抗菌治疗提供了无抗生素的替代路径。值得强调的是，我们还发现，这些高亲和力的人工受体不仅可实现靶向识别，还能诱导细菌之间的聚集，激活群体感应系统中受调控的信号分子表达，进而促进细胞工厂中产物的合成效率提升。该研究工作系统展示了人工印迹受体在微生物识别、调控及工程化利用中的多功能潜力，为MIPs在合成生物学和生物制造领域的应用拓展提供了新思路与理论依据。

**图4:**
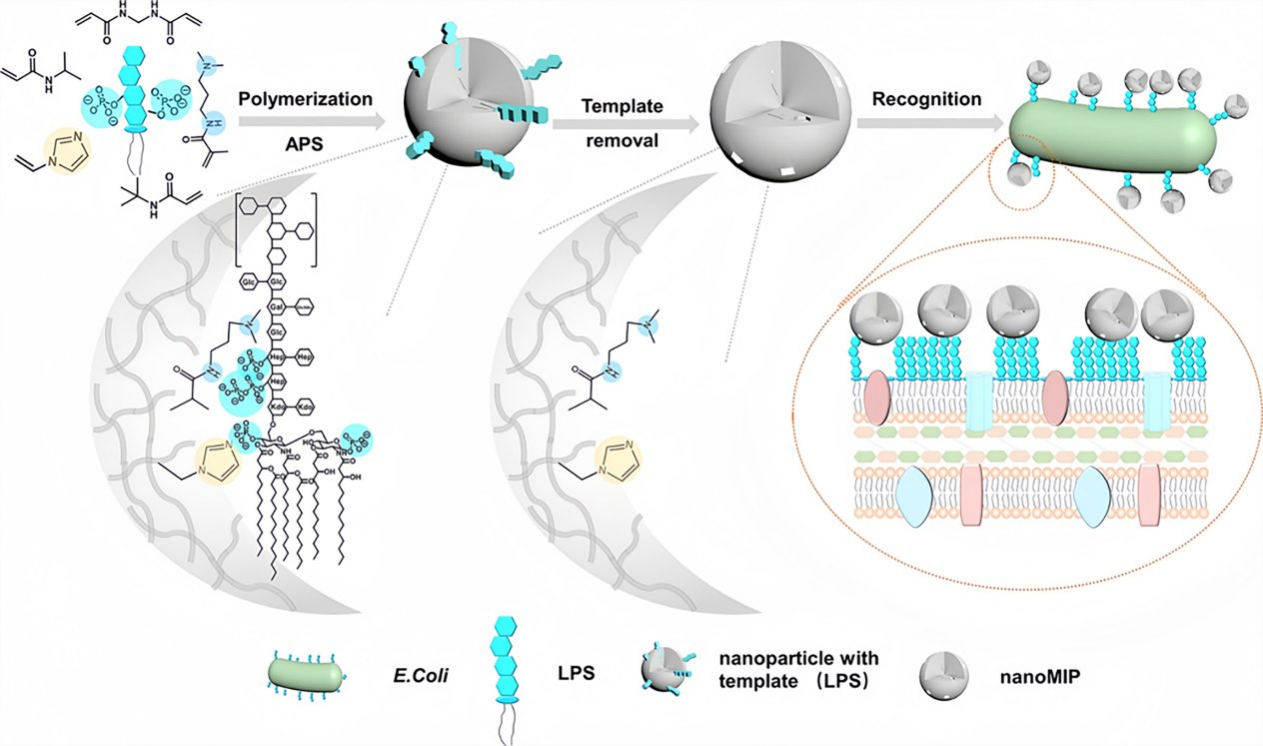
在LPS作为模板存在下，采用通过96孔板筛选出的最佳聚合物配方合成MIPs的示意图^［29］^

围绕细胞表面结构中的特异性组分的识别，分子印迹策略的创新性应用也在不断拓展深化。Liu团队^［30］^针对肝癌诊断中甲胎蛋白（AFP）的糖基化异质性，开发了“以一敌十”的双模态分子印迹策略：以双唾液酸化糖链A2G2S2为模板，通过硼酸亲和可控定向表面印迹技术构建MIPs，同步捕获含核心岩藻糖或唾液酸的AFP糖型（Gm）。结合表面增强拉曼散射（SERS）与激光解吸电离质谱（LDI-MS）双模态检测，实现了Gm/AFP比值的自验证分析，使肝癌诊断特异性显著提升至45%，相比传统ELISA方法的10%具有显著优势。该工作不仅整合了唾液酸化修饰的诊断价值，更为糖型分辨检测提供了新范式。在细胞表面磷脂标志物识别中，该团队^［31］^还提出油/水界面定向组装新策略：利用反相微乳液^［32］^将磷脂酰丝氨酸（PS）极性头定向暴露于界面，通过尿素功能单体（UPTES）形成氢键网络，构建出高选择性PS印迹材料。该MIPs可精准识别凋亡细胞表面外翻的PS，并成功应用于尿液外泌体的高效分离（效率较超速离心法提升73%），为液体活检提供了新工具。这些进展均凸显了MIT在解析细胞表面糖脂标志物中的独特优势，通过对模板分子与功能单体相互作用的理性设计，实现对柔性生物分子高保真识别，为疾病诊断和细胞调控提供了多样化解决方案。

### 2.3 固相模板印迹：构建高亲和性、可扩展分子印迹聚合物的新范式

为克服传统印迹材料中普遍存在的印迹位点分布不均、模板包埋及洗脱困难等问题，固相模板印迹（solid-phase imprinting）技术应运而生，并逐步成为提升MIPs性能的关键策略。该方法通过将模板分子预先固定于固相载体表面，配合高稀释度的功能单体在模板附近聚合，能够显著提高印迹位点的取向性与均一性，是本课题组长期探索并成功应用的关键技术路径之一^［33-35］^。

在具体实施方面，固相模板印迹常采用热响应型单体*N*-异丙基丙烯酰胺（NIPAAm）为骨架，与交联剂*N，N′*-亚甲基双丙烯酰胺（BIS）共同构建聚*N*-异丙基丙烯酰胺网络（PNIPAAm），同时辅以疏水性、酸性与碱性单体，实现多功能印迹位点的协同构建。值得一提的是，PNIPAAm具备32 ℃左右的下临界溶解温度（LCST），可在不同温度下切换亲疏水状态。这一热响应特性使得MIPs在合成后能通过温度调控实现模板分子的温和释放，极大简化了材料后处理流程。

相较于传统以玻璃珠为固相模板、MIP产率较低（约10 mg MIP/50 g玻璃珠）的体系，引入磁性纳米粒子作为载体后，单位载体质量的MIP产率提高至原来的100倍以上^［34，36］^。基于此平台，本课题组^［33］^构建了一种可靶向心衰标志物B型利钠肽（BNP）的荧光MIPs传感系统（图5）。该系统通过引入荧光素丙烯酰胺作为示踪单体，实现纳米颗粒荧光标记，同时筛选出高亲和力（*K*_D_为2.47×10^-11^ mol/L）和高选择性MIPs。最后，包裹量子点（CD）的MIP纳米粒子（nanoMIP@CD）被成功制备，用于实现对人血清样本中BNP的定量检测^［33］^。当nanoMIP@CD选择性地与血液样本中的BNP相互作用时，捕获的BNP可渗透进入交联聚合物网络中，与CD近距离接触。而CD和BNP之间的电子转移会导致CD的直接荧光猝灭，成功实现血清样本中BNP在7 min内的超灵敏检测（检出限为0.208 pg/mL，线性范围为0.25~5 000 pg/mL）。在160例临床血清样本检测中，该方法检测结果与电化学发光法高度一致（相关系数为0.994），充分验证了其在真实临床样本中的诊断潜力（图6）。

**图5:**
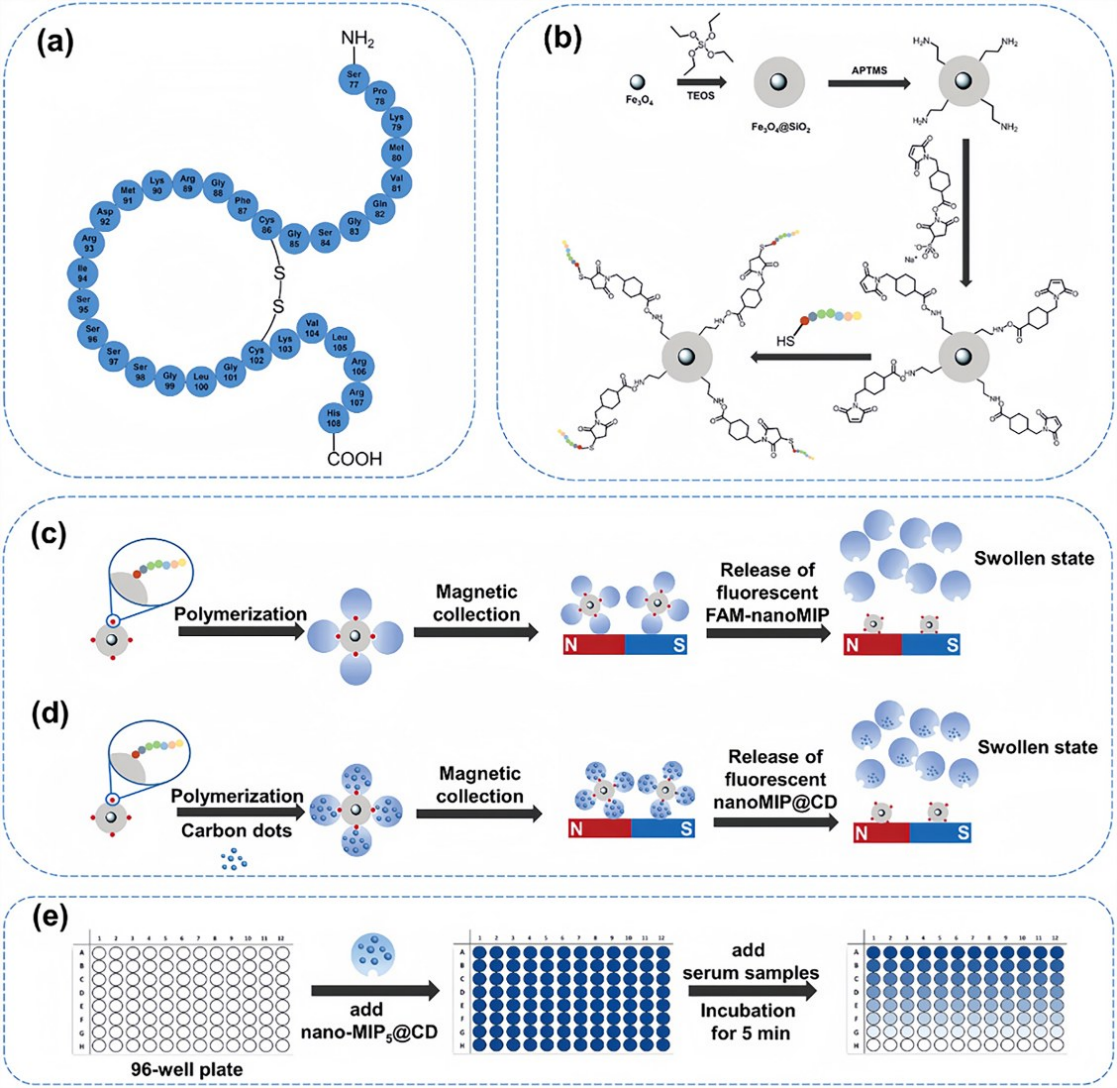
（a） BNP的氨基酸序列结构； （b）通过将表位多肽固定在功能化的磁性纳米颗粒上制备磁性模板的过程示意图； （c）5-羧基荧光素修饰MIPs纳米颗粒的合成过程； （d） MIPs包被碳量子点的合成过程； （e）荧光法检测血清样品中的BNP^［33］^

**图6:**
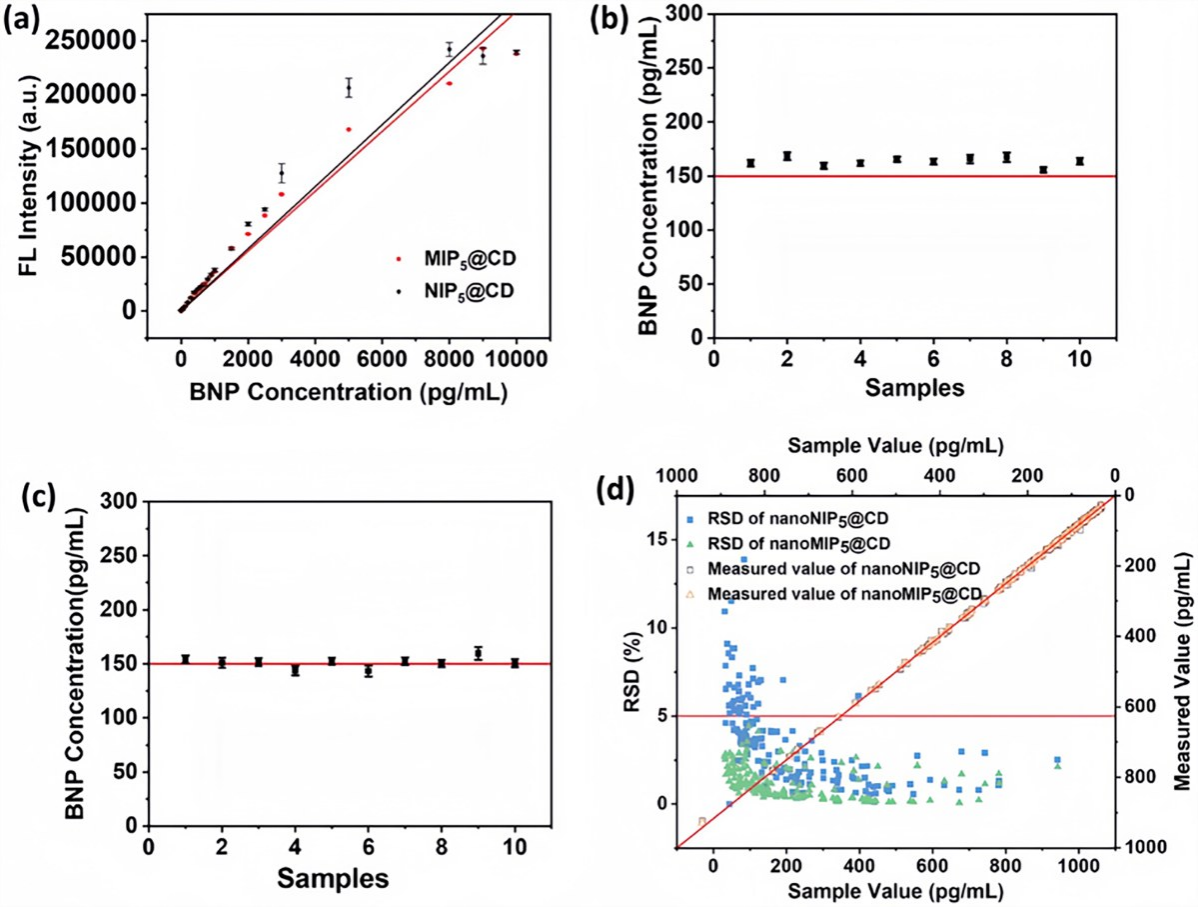
（a）基于BNP浓度与荧光强度函数关系的分析校准曲线；（b）荧光检测法重现性研究：采用10个独立合成批次的nanoMIP@CD测定同一加标BNP的血清样本；（c）方法重复性研究：使用同批次nanoMIP@CD测定10份等浓度加标BNP的血清样本；（d）基于nanoMIP@CD/nanoNIP@CD的荧光检测法与临床常规电化学发光法对160例未知血清样本的BNP检测结果对比^［33］^

同类研究进一步拓展了固相印迹技术的应用维度。Cai团队^［37］^利用固相印迹策略成功构建了针对甲型流感病毒特定亚型的单克隆分子印迹传感器，展现出对目标病毒颗粒的高亲和力特异性识别能力。该传感器可以从高度相似的甲型流感病毒亚型（例如H1N1、H7N9和H9N2）中识别出目标病毒，检出限为0.58 fmol/L。另外，该团队^［38］^基于“点对点”帽状结构创新性地设计构建了双功能分子印迹传感器，突破了单靶标检测局限，实现了对甲型肝炎病毒（HAV）和乙型肝炎病毒（HBV）的同时、高选择性检测（对HAV和HBV的检出限分别为27.1 pmol/L和34.3 fmol/L），解决了相似病毒亚型同步检测的难题，为临床多病毒筛查提供新工具。同样基于固相印迹理念，Sellergren团队^［39］^通过精心设计模板固定与功能单体组装，合成了高选择性MIPs（印迹因子为9），其荧光“开启”响应特性为磷脂分子的灵敏、特异性识别提供了新手段。这些研究共同彰显了固相印迹技术在构建复杂生物靶标（病毒、磷脂）高选择性识别界面方面的显著优势与广阔应用潜力。

此外，固相模板印迹法亦适于高通量并行合成。基于此特性，我们进一步扩展了其在肿瘤细胞识别领域的应用^［34］^。通过合理设计前列腺特异性膜抗原（PSMA）的表位多肽作为模板，并结合磁性固相模板，我们构建了荧光标记的印迹纳米凝胶（MIP-M），其对PSMA表位多肽与完整蛋白分别表现出*K*_D_=6×10^-10^ mol/L和2.5×10^-9^ mol/L的高亲和力（图7）。相较于多肽和人血清白蛋白（HSA），MIP-M显示出优异的选择性。通过流式细胞术和共聚焦激光扫描显微镜比较表达正常（PC3细胞）和增强（LNCaP细胞）PSMA水平的细胞系，结果显示MIP-M对后者的结合偏好与PSMA抗体相当。此外，在选择性靶向PSMA阳性前列腺肿瘤组织（而非正常组织）方面，MIP-M的性能也与PSMA抗体相当。这种MIP-M有效解决了以往靶向剂在稳定性、生产难度、特异性及毒性方面的问题，为PSMA导向的癌症诊断和治疗提供了一个极具前景的替代方案。我们所采用的模板固定控制及分散聚合方法，有望推动MIT在肿瘤成像等生物医学领域的应用发展。

**图7:**
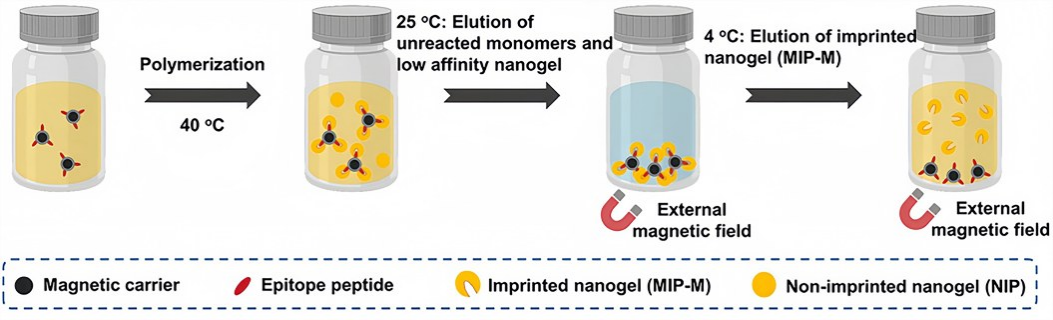
使用磁性固相模板介导的分散相印迹方法制备表位印迹纳米凝胶（MIP-M）的过程示意图^［34］^

固相印迹与功能性材料的融合成为癌症治疗等方面的研究趋势：Liu团队^［40］^将光热转换材料（如金纳米棒）集成至固相印迹材料中，使MIPs在近红外照射下同时实现目标识别（程序性死亡配体-1（PD-L1）蛋白）和局部热疗，肿瘤抑制率达74.2%。该进展突破了传统固相印迹的静态识别局限，赋予材料环境自适应能力。同时，固相印迹技术的应用正加速向治疗干预领域拓展。Sabbah团队^［41］^利用固相印迹策略成功制备了靶向滋养层细胞表面抗原2（TROP2，在多种侵袭性乳腺癌中高表达）的MIPs。该MIPs能特异性识别并高效结合乳腺癌细胞表面的TROP2蛋白。通过负载化疗药物紫杉醇（PTX），构建的TROP2-MIP@PTX递送系统在体内外均展现出显著的抗肿瘤活性：在体外能选择性杀伤TROP2高表达的乳腺癌细胞；在小鼠模型中，相比游离PTX和非靶向纳米粒，TROP2-MIP@PTX显著抑制了肿瘤生长并延长了生存期，凸显了固相印迹材料作为靶向药物递送平台的巨大潜力。另外，固相印迹材料在调控细胞命运方面亦展现出独特价值。例如，Domingues团队^［42］^将转化生长因子-β3（TGF-β3）表位印迹纳米颗粒整合至生物工程支架中，精确调控局部微环境中TGF-β3的浓度和生物利用度。这些“功能性”颗粒能持续、局部地提供TGF-β3信号，协同支架的物理拓扑结构（如排列的纳米纤维），有效引导间充质干细胞向肌腱细胞分化，显著上调肌腱相关基因（如SCX， TNMD， COL1A1）和蛋白的表达，为功能性肌腱组织再生提供了新策略。这些研究有力拓展了固相印迹技术在复杂生物样本检测、精准靶向治疗及组织工程调控等场景的多维应用潜力。

分子伴侣通过选择性结合非天然态蛋白质来辅助其重折叠。尽管人工分子伴侣的设计已取得一定进展，但其适用底物范围仍然有限。基于固相模板印迹技术，本课题组提出了一种可定制的仿生分子伴侣——分子印迹柔性聚合物纳米粒子（nanoMIPs）^［35］^。我们以细胞色素c、漆酶和脂肪酶等模型蛋白为代表，筛选了多种聚合单体，确定了最佳配方，使其具备可调节的电荷和疏水性。采用固相模板印迹技术，以固定变性全蛋白的磁珠作为固相模板，通过磁分离方式实现反应介质的更换，从而高效合成出具有印迹位点、模拟分子伴侣腔体结构的nanoMIPs。这些nanoMIPs能够选择性地识别并重折叠变性酶，最高可实现86.7%的酶活性恢复，显著优于对照组。机制研究证实，nanoMIPs优先结合变性酶而非天然态酶，模拟了天然分子伴侣的识别机制。多维分析结果表明，这些nanoMIPs可通过选择性结合变性蛋白质，抑制其聚集并促进其重折叠，从而有效发挥类分子伴侣的保护作用。该策略在生物催化和生物医药等领域的蛋白质修复和回收中展现出广阔的应用前景（图8）。

**图8:**
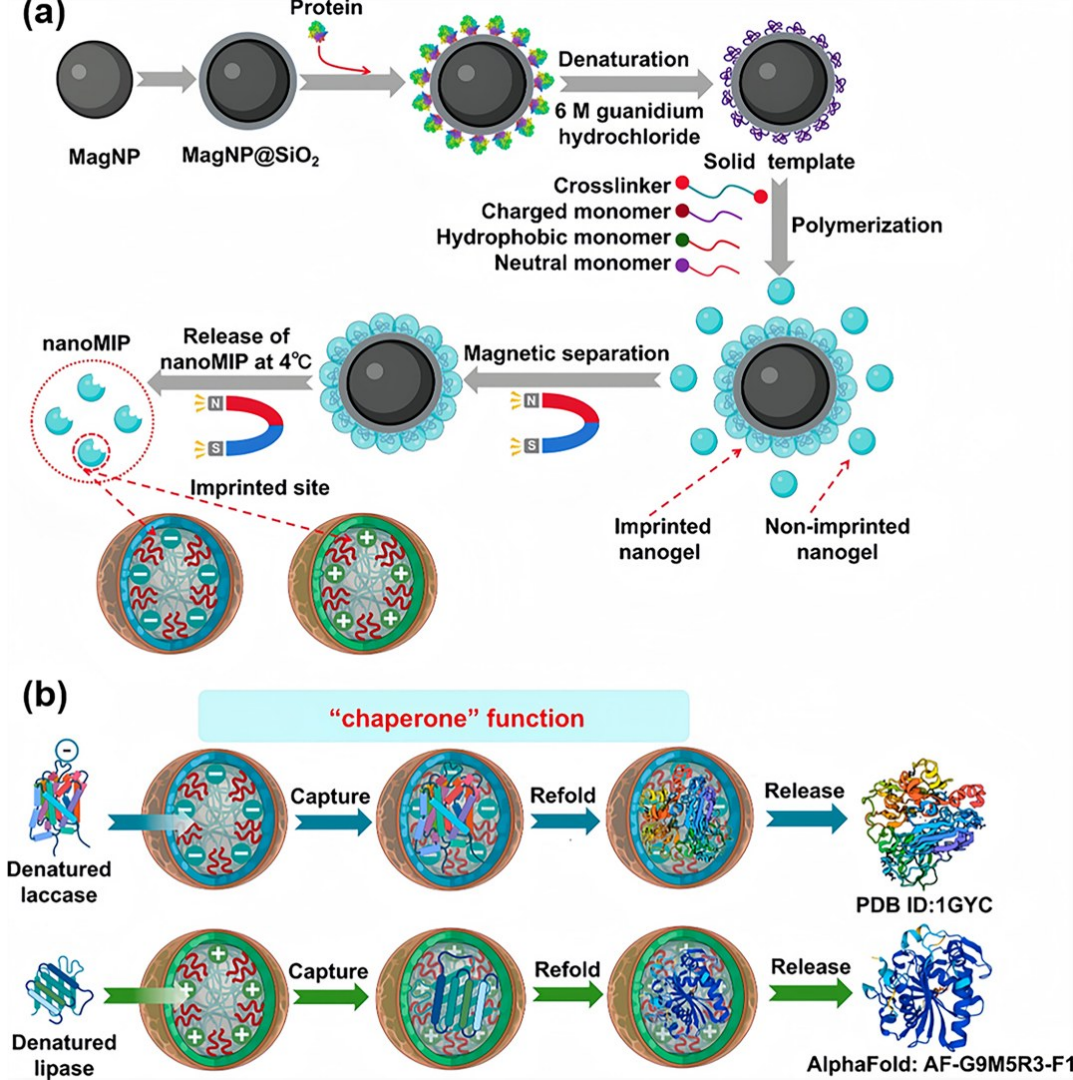
（a）将蛋白固定在磁性纳米颗粒上，用盐酸胍进行变性处理，合成、富集和纯化nanoMIPs的过程示意图；（b）使用nanoMIPs恢复变性酶的过程示意图^［35］^

令人瞩目的是，固相印迹技术在酶功能调控方面的应用已超越单纯的酶活性恢复或抑制，正深入拓展至病理细胞进程的精准干预。利用金属配位辅助的固相印迹策略，Pan团队^［43］^构建了高特异性、强效的分子印迹酶抑制剂（MIEIs），分别靶向胰蛋白酶（trypsin）和血管生成素（一种促癌的核糖核酸酶，RNase），其抑制常数（*K*_i_）低至纳摩尔级（如胰蛋白酶抑制剂*K*_i_=3.4 nmol/L）。尤为重要的是，该研究实现了MIEIs从体外简单的酶活性抑制向体内细胞命运调控的跨越：胰蛋白酶抑制剂（MIP_trypsin_）能有效阻止胰酶消化诱导的细胞外基质（ECM）溶解和肝细胞损伤，保护效果优于天然抑制剂α1-抗胰蛋白酶；而针对RNase的抑制剂（MIP_RNase_），通过嫁接黏蛋白1核心肽（MUC1）适配体实现癌细胞靶向，在进入人宫颈癌（HeLa）细胞后不仅能有效抑制其核糖核酸酶活性（半数抑制浓度（IC_50_）为6 nmol/L），更能显著降低细胞内外的RNase水平，最终成功抑制癌细胞增殖，效果优于天然核糖核酸酶抑制剂，且作用持久。这一突破性进展充分展示了固相印迹技术所制备的MIEIs在治疗酶依赖性疾病（如组织损伤、癌症）方面的巨大潜力，标志着其在从分子识别工具向细胞功能调控治疗剂演进的关键一步。

## 3 分子印迹聚合物在生物医学与生物催化领域的多功能应用

MIPs在蛋白质亲和分离和纯化中的应用凸显了其在分子印迹领域的重要价值，而分子印迹整体柱（MIMs）作为兼具高选择性识别与快速传质能力的材料平台，也因此在相关研究中广受关注^［44］^。早期MIMs主要通过原位热引发自由基聚合反应制备而成：将模板分子、引发剂、功能单体与交联剂溶解于成孔溶剂中，注入不锈钢柱后完成聚合，并以乙腈洗脱除去模板与成孔剂，最终在聚合物内部形成特定的印迹空腔^［45］^。随着技术进步，紫外光或发光二极管（LED）光源引发的光引发聚合法被引入该体系，避免了高温条件下可能对蛋白模板造成的损伤，显著提升了MIMs的合成效率与模板适配范围^［46，47］^。

此外，表面引发的原子转移自由基聚合（ATRP）技术为MIMs功能化带来了全新契机。一方面，ATRP具备精确控制聚合链长和结构的优势，能够实现高性能MIPs的构建；另一方面，该方法适用于制备响应外部刺激（如温度、pH、离子强度等）的“智能型”材料。本课题组^［44］^基于ATRP合成了具备热敏响应能力的MIMs（图9）：在乙烯化毛细管内先合成聚（甲基丙烯酸缩水甘油酯-共-乙烯基二甲基丙烯酸酯）骨架，通过环氧基团与乙二胺反应引入氨基，随后接枝NIPAAm单体，构建热响应印迹聚合物层。该MIMs对其模板肌红蛋白（Mb）展现出优异的选择性识别能力，最大吸附容量达1 641 mg/g，并可在温度变化条件下实现蛋白质的高效捕获与释放。其温度响应机制源于低交联PNIPAAm层的热敏特性：当温度低于其LCST（32 ℃）时，PNIPAAm链呈亲水伸展状态，印迹空腔充分暴露，利于蛋白质的扩散与结合；当温度升至LCST以上，PNIPAAm链发生疏水塌缩，导致印迹空腔构象收缩并产生“挤出力”，促使蛋白质从空腔中释放。这一可逆的构象切换实现了对蛋白质的温和、非破坏性控释^［8］^，无需依赖有机溶剂或剧烈的化学反应。

**图9:**
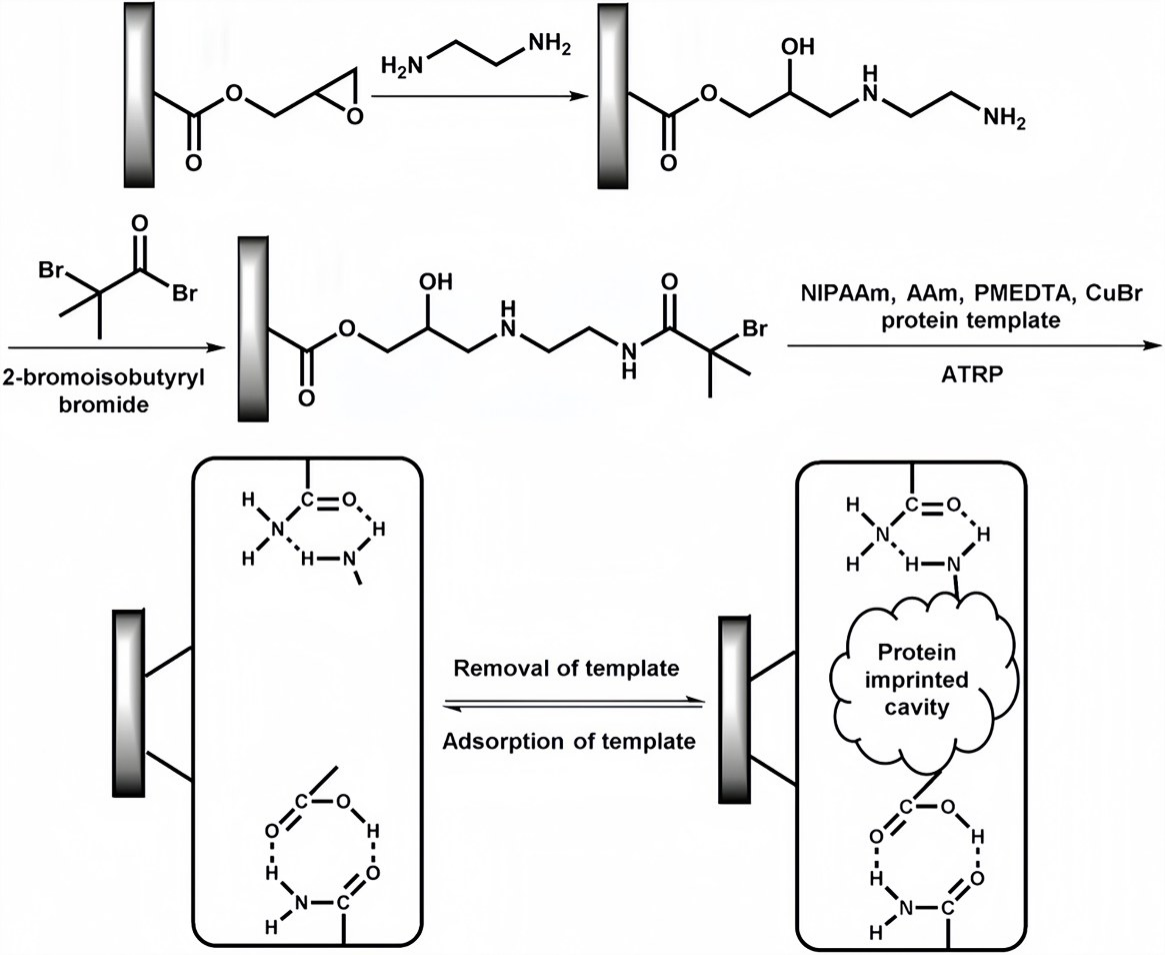
聚（甲基丙烯酸缩水甘油酯-二甲基丙烯酸乙二醇酯）整体柱的表面改性及蛋白印迹PNIPAAm层接枝合成方法（该过程包括利用乙二胺和2-溴异丁酰溴进行表面官能化，随后引发聚异丙基丙烯酰胺（PNIPAAm）层的表面接枝聚合）^［44］^

在pH响应体系中，我们进一步拓展了ATRP策略的应用边界。以pH敏感聚合物聚（4-乙烯基苯硼酸）（PVPBA）为功能层，在整体柱基底上接枝印迹层，靶向识别含顺式二酚基团的糖蛋白^［48］^。该体系成功实现了辣根过氧化物酶（HRP）与葡萄糖氧化酶（GOx）的共固定，构建出具备可逆酶修复能力的双酶系统：通过调控pH可实现酶的可控剥离与再加载。其响应机制源于苯硼酸基团与糖蛋白顺式二醇的可逆共价结合特性：在碱性条件（pH>8.5）下，苯硼酸电离为硼酸根阴离子，与邻二醇形成稳定的五元环酯键，实现酶的特异性捕获；当pH降至酸性环境（pH<6.5）时，硼酸根质子化导致酯键水解，促使酶从印迹位点释放。该动态共价键的可逆切换，结合PVPBA链在酸碱环境中的亲疏水性变化，协同实现了酶的高效、温和剥离与再加载^［49］^。该体系表现出广泛的线性检测范围（0.11~38.85 mmol/L）和优异的灵敏度（检出限为0.03 mmol/L），并在实际人血清样本中展现出良好的应用前景。

MIT在酶固定化与酶活性调控等生物催化领域的应用，体现了技术与需求之间的双向反馈机制。本课题组从最初关注结构稳定性与活性保持的“全蛋白印迹”，到后续通过尺寸调控实现构象保护的MOFs印迹系统^［27］^，再到最新以“分子伴侣”为理念的固相印迹纳米材料^［35］^，均展现出MIPs技术对酶性能调控的逐步深化。值得关注的是，分子印迹技术正推动酶抑制剂设计范式的革新：通过精准模拟酶变构位点开发的新型印迹抑制剂，不仅能高效阻断靶酶活性，更可深入调控病理细胞进程。这种“酶活性抑制-细胞功能干预”的双重调控机制，标志着MIT介入疾病发生的分子通路调控领域的发展，为开发智能型治疗剂提供了新范式^［43，50］^。

在仿生抗体方向，MIPs通过模拟抗原-抗体识别界面，实现了无需生物免疫过程的高特异性结合。例如，通过降低交联密度和引入溶胀水凝胶网络，研究者陆续开发了一系列对蛋白模板具有智能响应能力的水凝胶型MIPs；这些MIPs不仅保持了识别性能，还具备高效模板释放能力，拓展了其在疾病早筛和癌细胞成像等领域的应用^［51，52］^。Pan团队^［53］^构建了温敏分子印迹水凝胶，利用温度调控其唾液酸识别能力，实现了从全血中选择性分离癌细胞；Xu等^［54］^创新设计了两类仿生识别元件，适配体/MIPs，结合电化学传感器、微流控芯片及人工智能技术，实现了循环肿瘤细胞（CTCs）的高效捕获（多价适配体将捕获效率提升39倍，MIPs水凝胶达94.7%）、无损释放（细胞活性>86%）与精准检测（检出限低至1 细胞/mL）。为了更好地解决CTCs捕获过程中选择性与细胞活性保持的难题，Zhang团队^［55］^结合细胞印迹技术（物理结构匹配）和硼酸盐亲和（化学识别），以人肝癌细胞SMMC-7721为模板，制备了表面具有精准微纳结构并修饰3-丙烯酰胺基苯硼酸（3-AAPBA）的MIP水凝胶。该材料通过协同效应实现了高效捕获（90.3%捕获率）和高选择性识别（在千倍非目标细胞干扰下富集因子达13.5）SMMC-7721细胞，并能在生理条件下近乎完全（99%）无损释放细胞，且释放的细胞保持增殖能力。随后，在真实血液样本中的捕获效果（捕获效率49.9%）进一步证明了其在临床CTCs分离与分子分析中的应用潜力。而Qing团队^［56］^则设计出特异性靶向CTCs表面唾液酸化聚糖的双组氨酸（HH）单体（亲和力*K*_D_值为5.41×10^-6^ mol/L），并将其与聚乙二醇二甲基丙烯酸酯（PP）结合，同样以SMMC-7721细胞为模板制备MIPs水凝胶。该MIPs具备低溶胀率（19.8%）、高弹性、优异血液相容性和抗污染能力，实现了超高效CTC捕获（>95%捕获率，低至10个细胞时仍达93%）及无损动态释放（胰蛋白酶-EDTA溶液处理）；临床验证中，该技术成功区分了AFP<20 ng/mL的肝癌、肝硬化及健康人群，诊断准确率达94%。前沿研究正积极推动MIT在肿瘤液体活检等精准医疗方向的功能创新与应用深化。磷脂是肿瘤细胞表面一类重要的特有的受体或分子标志物，Sellergren团队^［57］^通过可逆加成-断裂链转移（RAFT）聚合法，以脂质鞘氨醇-1-磷酸（S1P）及其受体调节剂芬戈莫德磷酸盐（FP）为模板，将兼具识别与荧光信号功能的硝基苯并噁二唑-脲单体与交联剂接枝于二氧化硅核表面，制备了核壳结构MIPs（壳层厚10~20 nm）。该传感器在水性缓冲介质和稀释人血清中均表现出快速响应特性（约10 min），且对S1P和FP具有选择性荧光调节能力（S1P诱导荧光猝灭，FP诱导荧光增强）。在甲醇-水（50∶50）体系中，该传感器对S1P的检出限为5.6 μmol/L（线性范围为18~60 μmol/L），接近其生理浓度范围（约1 μmol/L），并成功应用于加标人血清样本的定量分析，为磷脂实时监测提供了新型传感平台；Liu团队^［58］^结合了SERS检测、基于碱基配对的细胞内微萃取技术以及硼酸亲和MIPs标签，实现了对目标tRNA的特异性捕获和修饰位点的选择性标记。该方法实现了在单细胞尺度观测氧化应激下tRNA修饰水平的变化，其灵敏度（检出限达1 pmol/L）和操作便捷性（约30 min）显著优于传统方法（如液相色谱-质谱），为探索tRNA的生物学功能提供了强有力的工具。这些研究均显著提升了分子印迹材料在复杂生物样本（如血液）中对稀有目标（如CTCs、特定膜脂、RNA修饰）的识别灵敏度、选择性和操控能力（如无损捕获与释放），为肿瘤的早期诊断、实时监测及后续分析提供了强有力的技术支撑^［59］^。

在肿瘤的免疫治疗领域，特别是在阻断免疫检查点介导的免疫抑制方面，MIPs正展现出强大的干预能力^［60］^。Liu团队近年来致力于开发精准靶向关键免疫调控分子的“合成抗体”型MIPs。例如，该团队构建了可特异性阻断血管内皮生长因子（VEGF）信号通路的分子印迹纳米药物，有效抑制肿瘤血管生成，为抗血管生成治疗提供了新策略^［61］^。更引人注目的是针对程序性死亡受体-1（PD-1）/PD-L1蛋白MIPs的开发^［62］^，这些MIPs能够高亲和力、高选择性地结合并阻断PD-L1，从而解除其对T细胞的抑制作用，重激活抗肿瘤免疫应答。为进一步提升疗效，该团队^［40］^还创新性设计了“分子印迹溶酶体纳米降解剂”，其不仅能阻断PD-1/PD-L1相互作用，更能主动将肿瘤细胞表面的PD-L1靶向递送至溶酶体进行高效降解，显著逆转肿瘤微环境的免疫抑制状态，在小鼠模型中实现了高达74.2%的肿瘤抑制率。这些突破性进展标志着MIPs已从单纯的识别工具跃升为能够主动干预关键免疫通路、重塑肿瘤免疫微环境的智能治疗剂，为开发下一代肿瘤免疫疗法开辟了极具前景的非生物抗体路径^［63-65］^。

在此基础上，Liu团队持续深化在该领域的研究，不断拓展MIPs的治疗维度与策略深度。针对乳腺癌治疗性抗体具有耐药性的问题，他们^［66］^构建了分级结构分子印迹纳米转换器（MINT），其核心为上转换纳米粒子（UCNP），外壳为薄层MIPs，表面修饰光敏剂Ce-6。MINT利用印迹的截短人表皮生长因子受体（p95HER2）N端表位特异性靶向耐药乳腺癌细胞，并通过近红外光（980 nm）激发UCNP，将能量转移至Ce-6产生活性氧（ROS）杀伤肿瘤。实验证实，MINT在体外和体内均能精准识别p95HER2^+^癌细胞，高效生成ROS，显著抑制肿瘤生长（体内抑瘤率达86.9%），为靶向突变耐药癌症的光动力治疗提供了新策略。在神经母细胞瘤治疗中，该团队^［67］^利用硼酸亲和定向表面印迹技术制备出特异性识别肿瘤细胞表面过度表达的聚唾液酸（PSA）的MIPs，并负载光热剂吲哚菁绿（ICG）形成“纳米导弹”。该纳米系统在体外和体内均能精准靶向PSA高表达的神经母细胞瘤细胞，在808 nm激光照射下快速产生局部高温（8 min内升温至65 ℃），实现高效光热消融，显著抑制肿瘤生长，同时展现出良好的生物相容性和稳定性，为神经母细胞瘤的靶向治疗提供了新平台。此外，该团队^［68］^还以磁性Fe_3_O_4_为内核、唾液酸MIPs为外壳，通过磁靶向富集于肿瘤部位并特异性结合肿瘤细胞过表达的唾液酸，标记肿瘤细胞为“异物”，从而激活巨噬细胞的吞噬作用，调控肿瘤相关巨噬细胞（TAMs）表型，通过诱导级联效应激活抗肿瘤免疫应答，显著提升了免疫治疗效果^［69］^。最终，在4T1乳腺癌模型中实现了53%的肿瘤生长抑制，且不影响正常组织。他们的研究^［70］^还聚焦于克服胞内递送屏障，设计氧化还原响应的MIPs。具体而言是将细胞毒性蛋白核糖核酸酶A（RNase A）封装于二硫键交联的二氧化硅纳米颗粒中（包封率为64%），并通过表面分子印迹技术赋予其靶向癌细胞表面过表达唾液酸的能力。该纳米载体在生理条件下稳定，并能在肿瘤细胞内高浓度谷胱甘肽（GSH）的还原环境下发生生物降解，实现蛋白质的控释。体外实验证实其可选择性靶向肿瘤细胞，促进细胞摄取，并在细胞内快速释放RNase A，显著增强细胞毒性。体内实验进一步证明其在异种移植瘤模型中具有特异性肿瘤靶向能力和高效治疗功效，显著抑制肿瘤生长（抑瘤率68.5%）。该研究成功实现治疗性蛋白质向癌细胞的靶向递送与可控释放，解决了治疗性蛋白质在癌症治疗中面临的易降解、膜渗透性差等递送难题，为基于蛋白的癌症治疗提供了新方法。综上，这些研究从克服耐药、开发新靶点、增强治疗效力、激活免疫响应到实现精准胞内递送等多个层面，共同展现了MIPs作为智能递送平台和效应分子在解决现有癌症免疫治疗挑战方面的巨大潜力与创新活力^［71，72］^。

此外，MIPs在构建智能诊疗一体化载体方面展现出持续创新。例如，Zhang团队^［73］^构建了以阿霉素（DOX）负载的荧光沸石咪唑酯骨架-8（FZIF-8）为核心、CD59糖蛋白表位印迹的MIPs为外壳的FZIF-8/DOX-MIP纳米颗粒。该载体通过MIP表位印迹腔特异性识别人乳腺癌MCF-7细胞膜过表达的CD59糖蛋白实现肿瘤靶向富集，同时利用MIP骨架中的二硫键交联剂（响应高浓度GSH）和pH响应单体（响应弱酸性微环境），在肿瘤微环境下发生降解，暴露内部的FZIF-8/DOX并进一步释放DOX。体外实验证实其对MCF-7细胞具有选择性吞噬和杀伤效果，体内实验显示其能精准靶向肿瘤部位实现荧光成像，并在异种移植瘤模型中显著抑制肿瘤生长。这类基于印迹材料的策略巧妙结合了精准识别能力与刺激响应性载药设计，为克服传统纳米药物靶向效率不足、释放不可控等难题提供了新方案，有力推动了诊疗一体化平台向临床应用迈进^［74］^。

本课题组也进一步拓展了MIPs作为仿生受体的应用，使其具备光热灭菌及群体感应调控等多重功能。在细胞工厂系统中，MIPs通过调节细菌的聚集行为与信号感应机制，有效提升了代谢通量，彰显了其在合成生物学领域的独特潜力与应用价值^［75］^。

此外，我们还构建了基于非印迹聚合物的酶抑制剂^［76］^，通过在聚合物结构中引入苯酚基和苯硼酸基两种功能团，实现了对酪氨酸酶的协同抑制。其中，苯酚基可与酶的催化铜离子发生配位作用，而苯硼酸基则能与底物邻苯二酚发生竞争性结合，两者协同作用可显著降低酶活性。该策略在体外和体内均成功抑制了酪氨酸酶活性与黑色素的生成，验证了其优异的抑制性能。该系列研究不仅展示了MIPs及其衍生材料在复杂生物系统中的功能拓展，也为构建基于非生物高分子的新型酶抑制剂提供了创新设计路径，拓宽了分子识别与调控在酶工程及疾病治疗等领域的研究边界。

## 4 总结与展望

近年来，分子印迹技术在生物医学和生物催化领域展现出显著的应用潜力。本文系统梳理了本研究团队近5年来在MIT领域的前沿探索，结合国际领先研究，尤其是MIT在疾病诊疗与生物催化中的新兴应用，全面总结了理性分子设计、功能材料构建、生物传感与诊疗一体化等方向的研究进展。

本团队依托固相印迹平台与计算模拟辅助设计策略，系统解决了大分子印迹中模板构象稳定性、残留物高效去除以及水相环境适配性等关键科学难题，推动分子印迹材料从传统的静态识别载体向具有智能响应能力的生物医学工具升级。

展望未来，为实现MIT在临床转化中的关键跨越，我们认为需重点突破以下三大前沿方向：（1）构建新一代智能响应材料体系。未来MIT将向多模态、可编程方向演进，重点开发对特定生理微环境具备特异响应能力的印迹材料，特别是对磁、光、热等外部刺激具有协同响应的多功能体系。通过引入动态可调控机制，可实现精准递药、远程激活与实时信号监测，推动印迹材料从被动识别向主动调控与个体化治疗转变。（2）建立动态识别机制的理论框架与预测模型。当前MIPs的设计多基于静态模板，缺乏对识别过程的动态响应与能量演化规律的系统解析。未来应结合人工智能与多尺度模拟技术，量化研究印迹空腔柔性调节与靶标分子构象变化之间的耦合机制，构建从分子到体系的多尺度理论模型，以实现印迹识别行为的可预测调控，并指导自适应型印迹材料的理性设计。（3）构建闭环式集成智能诊疗平台。通过将微流控技术与仿生识别单元深度融合，开发集生物标志物检测、靶向药物递送与疗效实时反馈于一体的闭环诊疗系统，实现“体外精准检测-体内智能干预”的贯通式应用。该平台有望打通基础研究与临床实践的关键环节，推动MIT在个体化医疗与即时诊断中的落地应用。

分子印迹技术正处于从基础材料科学向复杂生命系统智能干预工具转化的关键阶段。本团队将持续推动固相印迹平台的标准化升级，联合跨尺度理论模型与实验验证，构建具有自主响应、动态调节与多功能集成的下一代智能MIPs系统。我们预期，随着其在个体化诊疗、即时检测设备与合成生物学平台中的不断扩展，MIT将实现从实验室技术突破向临床实际应用的深层转化，开启精准医疗的新篇章。
